# Dynamics of the transcriptomic landscape of OsHV-1 replication in haemocytes of the Pacific oyster (Magallana gigas)

**DOI:** 10.1099/jgv.0.002187

**Published:** 2025-12-15

**Authors:** Aurélie Dotto-Maurel, Jérémy Le Luyer, Nicole Faury, Lionel Dégremont, Margot Tragin, Tristan Renault, Benjamin Morga, Germain Chevignon

**Affiliations:** 1 ASIM Adaptation et Santé des Invertébrés Marins, Ifremer, F-17390 La Tremblade, France; 2 LEMAR, Univ Brest, Ifremer, CNRS, IRD, F-29280 Plouzané, France; 3 RBE, Département Ressources Biologiques et Environnement, Ifremer, F-44000 Nantes, France

**Keywords:** dual RNASeq, haemocytes, herpesvirus, *Magallana gigas*, ostreid herpesvirus type 1, oyster

## Abstract

Since the 1990s, the Pacific oyster (*Magallana gigas*) has experienced repeated mortality events associated with Ostreid herpesvirus 1 (OsHV-1). Although the virus has been genomically characterised, its replication cycle and its interactions with the oyster immune system are still not well understood. In particular, little is known about the dynamics of OsHV-1 gene expression and the immune responses of haemocytes from oysters with varying susceptibility to the virus. While some studies have focused on the expression of specific viral and host genes in whole oysters, none have provided a comprehensive analysis of genome-wide expression across multiple post-infection time points in haemocytes.

The lack of oyster cell lines makes studying virus–host interactions *in vitro* challenging. However, haemocytes, the key immune cells circulating in haemolymph, can be maintained *in vitro* in the short term and represent a relevant model for analysing infection dynamics. In this study, haemocytes from two *M. gigas* batches, one highly susceptible and one less susceptible to OsHV-1, were infected *in vitro*. We tracked the viral and host transcriptomes over a 24-h period post-infection using high-throughput dual transcriptomics.

Our results provide a detailed overview of the OsHV-1 transcriptomic landscape in haemocytes from high- and low-susceptible *M. gigas* over time. In addition, weighted correlation network analysis of host gene expression provided insights into the haemocytes’ response to infection and highlighted batch-specific immune responses. This comprehensive transcriptomic study is the first to describe virus–host interactions across multiple stages of infection in haemocytes from Pacific oysters, showing contrasted survival when exposed to OsHV-1.

**Impact Statement**

This study provides valuable insights into the interaction between Magallana gigas and OsHV-1 by analysing viral expression and host immune response at the cellular level. By focusing on haemocytes, the key immune cells in Pacific oysters, the results reveal a link between host susceptibility and viral transcriptomic activity, providing new perspectives on the molecular basis of natural susceptibility levels to OsHV-1 infection depending on the genetic background. Overall, our findings deepen the understanding of OsHV-1 gene expression dynamics and antiviral defence mechanisms in key species cultivated worldwide.

## Data Availability

All sequence data have been deposited in ENA under the accession number PRJEB88219. Detailed sample accessions are available in Table S5. Analysis scripts are publicly available at https://gitlab.ifremer.fr/asim/haemocytes-dualrna-seq-analysis.git. All supplementary materials are available at the following Figshare link: https://doi.org/10.6084/m9.figshare.30499793.

## Introduction

Herpesviruses are large, double-stranded DNA viruses known for their complex genome and biology [1]. Their viral life cycle alternates between a lytic phase, which is characterized by active replication and spread, and a persistence phase during which the virus remains dormant and evades detection by the immune system. Research focused on the lytic phase of human herpesviruses, particularly herpes simplex virus 1 (HSV-1) [2,3], showed that this phase involves a tightly regulated transcriptional cascade comprising three gene classes: (i) immediate-early genes, which initiate virion replication by activating early gene transcription; (ii) early genes, which code proteins necessary for viral DNA replication; and (iii) late genes, which produce virion structural components such as capsid proteins and glycoproteins [2,6].

While much attention has been given to herpesviruses that infect humans, these represent only 8 out of the 137 known herpesvirus species. The *Herpesviridae* family has a broad host range, infecting mammals, birds, fish, amphibians and marine molluscs [7,9]. In particular, since the 1990s, Ostreid herpesvirus 1 (OsHV-1) has emerged as a major pathogen in aquaculture, causing recurrent mass mortality events in Pacific oysters and resulting in significant economic losses. This herpesvirus, belonging to the family *Malacoherpesviridae*, is the only species in the genus *Ostreavirus* [10,11]. To date, several OsHV-1 strains have been characterized and sequenced [12,13]; C.-M [14,19]. Among them, OsHV-1 µVar is currently the predominant strain detected in oyster farming areas worldwide. Its genome spans ~207 kb and encodes 128 unique ORFs. While 55% of the predicted genes have no assigned function yet, the remaining genes are associated with processes such as secretion, membrane interaction, DNA replication and apoptosis inhibition [15,19].

The life cycle of OsHV-1 is not yet fully understood or characterized, but it is believed to follow the general pattern of other herpesviruses, involving alternating lytic and persistent phases [9]. When seawater temperature reaches and remains above 16 °C, OsHV-1 actively replicates and causes high mortality in juvenile oysters within 2 weeks in intertidal environments, and even more rapidly under laboratory conditions where oysters are continuously immersed [20,23]. Conversely, when seawater temperature falls below 13 °C, no mortality is observed, and OsHV-1 becomes largely undetectable, suggesting a transition to a persistent phase [20,21, 24, 25]. However, the persistence phase of OsHV-1 in Pacific oysters remains poorly characterized, and haemocytes are thought to contribute to viral maintenance, although the specific tissues or cell types involved are still unknown [26,27].

During the lytic phase, it has been suggested that OsHV-1 enters the Pacific oyster via the haemolymphatic system and then infects various organs throughout the haemolymph [28,29]. *In situ* hybridization studies and DNA detection by quantitative PCR (qPCR) have demonstrated that OsHV-1 can replicate in several oyster tissues, particularly in the gills, mantle, labial palps, digestive gland, gonad, heart, adductor muscle and ganglia [29,31]. Techniques such as reverse transcription real-time PCR, microarray and RNAseq have been used to study viral and oyster gene expressions [23,32,36]. Those approaches have shown that OsHV-1 gene expressions occur at an early stage in the infection and have enabled the classification of some OsHV-1 genes as either immediate-early or early [34,36]. While these findings have deepened our understanding of OsHV-1 gene expression dynamics during infection, they also raise important questions about the molecular mechanisms by which oysters detect and respond to viral invasion.

Antiviral immunity in mollusks is still not well understood. However, Pacific oysters have a sophisticated and well-coordinated defence system that can detect nonspecific nucleic acids and activate an antiviral response that inhibits subsequent OsHV-1 infection [23,37]. Moreover, it has been shown that OsHV-1 infection leads to increased expression of various genes associated with an interferon-like pathway, including viral recognition receptors, signal transducers, transcription factors and antiviral effectors [37,39]. However, while these molecular findings highlight the existence of a complex antiviral response in oysters, they do not fully explain the variability observed in disease outcomes between hosts with different genetic backgrounds.

Indeed, susceptibility to OsHV-1 infection varies significantly among Pacific oyster batches, suggesting that genetic background plays a crucial role in determining the effectiveness of the host immune response. While some batches are highly susceptible to OsHV-1 infection and experience high mortality rates, others show much higher survival rates [40]. Selective breeding has enabled the production of oyster batches that demonstrate contrasted susceptibilities to OsHV-1 infection [40,41]. Although low-susceptible Pacific oysters are not completely resistant to infection and still carry the virus, they are better at controlling viral replication than more susceptible ones [23,40]. Indeed, transcriptomic analysis showed that low-susceptible Pacific oysters exhibit a faster and more effective immune response than more susceptible ones. However, these analyses were conducted on whole-oyster tissue pools, which limits the ability to assess tissue-specific resolution of the immune response [32].

Currently, no cell line system is available to study OsHV-1 replication *in vitro*, making transcriptomic analysis of the lytic phase challenging. However, haemocytes, the circulating cells present in Pacific oyster haemolymph, can be maintained *in vitro* for several hours and can be infected by OsHV-1 to study the lytic phase within a simplified model [34]. Although haemocytes play a crucial role in the immune response of *Magallana gigas*, only a few studies have investigated their response to OsHV-1 infection [34,42].

Our study aims to provide a detailed overview of the OsHV-1 transcriptomic landscape in primary culture of haemocytes from low-susceptible (LS) and high-susceptible (HS) Pacific oysters over a 24-h infection period. RNA from haemocytes was extracted and sequenced using short-read technology to analyse viral expression and host response. This dual transcriptional analysis represents the first characterization of host–virus interactions in two phenotypically distinct oyster batches in terms of viral infection susceptibility over multiple infection times under *in vitro* conditions.

## Methods

### Pacific oyster production

One batch of *M. gigas* with high susceptibility to OsHV-1 (HS; 91% mortality at 11 days post-exposure) and another with low susceptibility (LS; 20% mortality at 11 days post-exposure) were conditioned at the Ifremer hatchery in La Tremblade in December 2014. The two batches correspond to the control and selected groups produced in the fourth generation of the line B described in a previous study [41]. The broodstock was maintained in 240-l raceways with a continuous supply of filtered seawater treated with UV and was richly fed with phytoplankton (*Isochrysis galbana*, *Tetraselmis suecica* and *Skeletonema costatum*) to promote gametogenesis. Before reproduction, 12 oysters per batch were tested for the detection of *Vibrio aestuarianus* and OsHV-1 using standard qPCR protocols [43,44].

Spawning occurred on 11 March 2015. For each batch, 25 to 30 oysters were placed in a 5-l beaker and induced to spawn using heat shock by changing the seawater temperature from 12–28 °C. Once spawning began, the seawater temperature was maintained at 25 °C. Gametes were sieved through a 100-µm sieve to remove larger tissue debris and then through a 20-µm sieve to remove small tissue/sperm debris. Fertilized and unfertilized eggs were retained on the 20-µm sieve. The embryos were then transferred to a 30-l tank with UV-filtered seawater, for which the temperature was maintained at 25 °C. Seawater was changed three times per week. Larvae were fed daily with *I. galbana*, *T. suecica* and *S. costatum* was added to the feed when larvae size exceeded 140 µm. Two weeks after fertilization, pediveliger larvae were transferred to sieves in 120-l tanks and raised under standard conditions in our controlled facilities (UV-treated seawater).

### Haemolymph collection

**Fig. 1.**
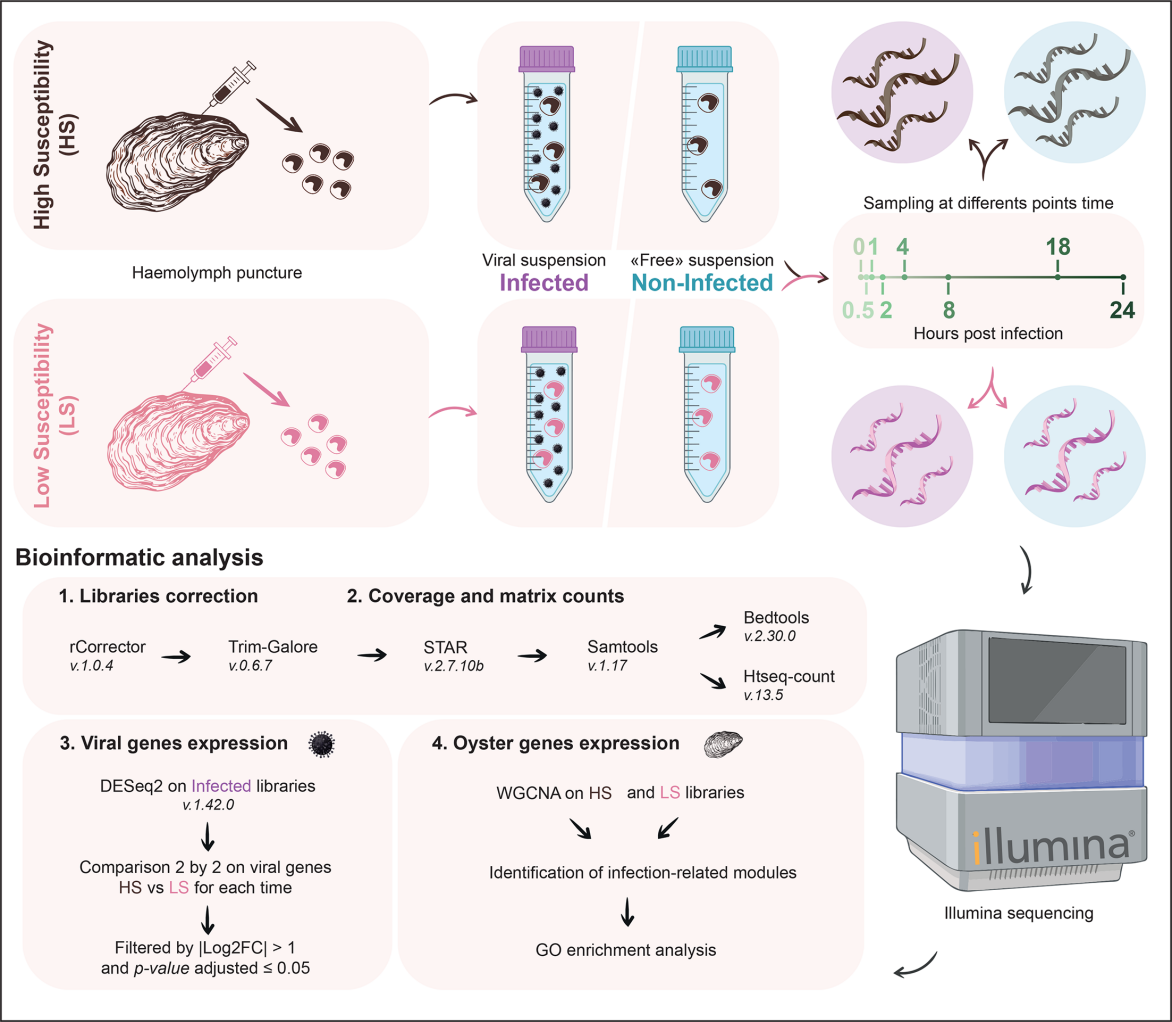
Experimental design and bioinformatic pipeline implemented in this study.

A total of 450 8-month-old oysters per batch were sampled for haemolymph collection (0.5–1 ml per oyster). Sampling was performed from the adductor muscle sinus using a 1-ml syringe fitted with a 20G AGANI™ needle (0.9×40 mm). To remove haemolymph debris, samples were filtered through a 60-µm nylon mesh and kept on ice to limit cell aggregation. Then, haemolymph samples were pooled within a batch, and haemocytes were counted using a Malassez cell. The haemocyte concentration was adjusted to 1.10^6^ cells per ml using filtered 0.22 µm artificial seawater (Fig. 1).

### Viral suspension

Viral suspension was prepared from infected oysters following the method outlined by Schikorski *et al*. [45]. To produce controls, the protocol described above was applied to oysters that tested negative for the detection of OsHV-1 DNA by real-time PCR to generate a virus-free suspension.

### *In vitro* infection

For each batch, the pool of haemocytes was separated into three replicates for each time and each treatment (i.e. infected and non-infected). The infected (Inf) haemocytes (1.10^6^ cells ml^−1^, 5 ml) were incubated with viral suspension (2.5 ml, 10^5^ copies of OsHV-1 µl^−1^) under gentle agitation at 19 °C and sampled after 0 min, 30 min, 1 h, 2 h, 4 h, 8 h, 18 h and 24 h of *in vitro* virus exposure (*n*=3 for each condition except at 30 min where *n*=2). Similarly, the non-infected (nI) haemocytes (1.10^6^ cells ml^−1^, 5 ml) were incubated using the virus-free suspension (2.5 ml) and the sample at the same time steps (Fig. 1).

To prevent fungal and bacterial growth in all samples, 350 µl of antibiotics was added, including 4 mg ml^−1^ of streptomycin, 11.6 mg ml^−1^ of penicillin, 5.1 mg ml^−1^ of neomycin, 3.3 mg ml^−1^ of erythromycin and 0.1 µL ml^−1^ of nystatin.

### Haemocytes DNA extraction and OsHV-1 DNA quantification

At each sample time, 7.5 ml of haemolymph was centrifuged for 10 min at 1,500 ***g***; the pellet was then DNA extracted using the QiAmp Tissue Mini Kit (QiAgen) following the manufacturer’s protocol.

To assess the viral genome copy number, a real-time qPCR using an Mx3005P thermocycler sequence detector (Agilent) was performed on extracted DNA.

Amplification reactions were performed in a total volume of 20 µl. Each well contained 5 ng of total DNA from oyster mantle or standard, 10 µl of Brilliant III Ultra-Fast SYBR®Green PCR Master Mix (Agilent), 2 µl of each primer OsHVDP For (forward) 5′-ATTGATGATGTGGATAATCTGTG-3′ and OsHVDP Rev (reverse) 5′-GGTAAATACCATTGGTCTTGTTCC-3′ [46] at the final concentration of 550 nM each and 1 µl of distilled water. For each qPCR plate, a five-point plasmid standard curve (ranging from 10⁵ to 10¹ OsHV-1 genome copies per ng of DNA) and a negative control were included. Real-time PCR cycling conditions were as follows: 3 min at 95 °C, followed by 40 cycles of amplification at 95 °C for 5 s and 60 °C for 20 s.

### RNA extraction and sequencing

Similar to DNA extraction, 7.5 ml of haemolymph was centrifuged for 10 min at 1,500 ***g****,* and the total RNA from the pellet was extracted using TRIZOL® Reagent™ (Ambion®) following the manufacturer’s recommendations. RNA was then treated with Turbo™ DNase (Ambion®) to remove any remaining DNA. After DNase treatment, a second RNA purification using TRIZOL was performed. The quality and quantity of the RNA were checked using NanoDrop. First-strand cDNA synthesis was carried out using the SuperScript® III First-Strand Synthesis System from Invitrogen with 500 ng of RNA. A No RT (no reverse transcription) control was performed after RNA extraction and was verified by qPCR to confirm the absence of oyster and/or virus genomic DNA using EF1*α* primers (targeting the elongation factor 1 alpha, Forward: 5′-GTCGCTCACAGAAGCTGTACC-3′, Reverse: 5′-CCAGGGTGGTTCAAGATGAT-3′) and OsHVDP For/OSHVDP Rev primers [targeting the ORF 100 coding for DNA polymerase: OsHVDP For (forward) 5′-ATTGATGATGTGGATAATCTGTG-3′; OsHVDP Rev (reverse) 5′-GGTAAATACCATTGGTCTTGTTCC-3′ [46]].

Sequencing of the 94 libraries (3 biological replicates per time point and condition, except for the 0.5 h post-infection (hpi) low-susceptibility batch, where 1 replicate failed due to insufficient mRNA quality) was carried out by Eurofins Genomics using strand-specific cDNA libraries sequenced on Illumina HiSeq 2500 and MiSeq platforms.

### Data analysis

All scripts described in this part are detailed in GitLab (https://gitlab.ifremer.fr/asim/haemocytes-dualrna-seq-analysis.git) and illustrated in Fig. 1.

### Annotation of the OsHV-1 genome

The experiment described in this study was carried out in 2015. For more accurate data analysis, we used an OsHV-1 reference as close as possible to the strain used at that time (raw data: SAMN18712119) [16], which was assembled from viral DNA extracted from infected oysters in the Bay of Marennes-Oléron in 2017 [16]. Considering the estimated mean evolutionary rate of 6.787×10⁻⁵ substitutions per site per year [16], only about 26 SNPs are expected between the reference genome and the viral genotype used in our experiments, which is unlikely to influence the results of our analyses.

This genome was then annotated using pairwise alignment with the OsHV-1 µVar A genome [15] using Geneious version 2025.0.2 (https://www.geneious.com). The annotation from the OsHV-1 µVar A genome was then transferred to the OsHV-1 genome used in this study. To refine the annotation, four ORF predictors were used: FragGeneScan [47], Vgas (K.-Y) [48], Prodigal (https://github.com/hyattpd/Prodigal) and GeneMark (https://exon.gatech.edu/GeneMark/gmhmme.cgi). Following prediction, ORFs that were added or modified relative to the OsHV-1 µVarA genome were labelled as ‘ORFS’ to indicate ORF (Table S1, available in the online Supplementary Material).

The functions and domains of all ORFs were then predicted using Interproscan version 5.73–104.0 [49] with all databases available and EggNogg mapper version 2.1.5–2.1.12 [50] web tools interrogated in February 2025. In addition, DeepLoc version 2.1 (https://services.healthtech.dtu.dk/services/DeepLoc-2.1/) was used to predict the subcellular localizations of viral proteins (Table S1).

### RNAseq read mapping, read count and descriptive statistics

The sequencing data were corrected using rCorrector [51] and Trim Galore (https://github.com/FelixKrueger/TrimGalore) with default parameters to remove adapters introduced during sequencing and to filter out short and poor quality reads (Script 01-Correct_libraries in GitLab). Reads were then aligned to the *M. gigas* mitochondrial genome (GenBank: GCF_963853765.1), the *M. gigas* nuclear genome (GenBank: NC_001276) and the OsHV-1 genome (raw data: SAMN18712119) [16] simultaneously, using STAR [52] with default parameters.

Forward and reverse count matrices were generated for each of the 94 datasets using HTSeq-count v.0.13.5 [53], with reads counted at the gene level using the -t exon, -i gene and -s yes or -s reverse options. All forward and reverse matrices were then combined to produce two complete count matrices covering all 94 datasets (Script 02-Alignments_Count_matrices). Outlier libraries were identified from the count matrix by plotting a heatmap and scatterplots adapted from the iDEP 2.01 scripts (http://bioinformatics.sdstate.edu/idep/).

Four different factors were then evaluated: (1) ‘treatment’ representing OsHV-1 infected or non-infected haemocytes, (2) ‘viral load’ corresponding to the amount of OsHV-1 genome copy number per nanograms of DNA, (3) ‘time’ which corresponded to the eight sample time points (0, 0.5, 1, 2, 4, 8, 18, 24 hpi) and (4) ‘batch’ traducing contrasted phenotype of the Pacific oysters: high- or low-susceptible to OsHV-1 infection.

To assess the relative contribution of each of these factors to the gene expression levels, a distance-based redundancy analysis (db-RDA) was performed. First, the ‘time’ factor was centred and scaled (sc). The data were then normalized using DESeq2 ‘median of ratios’ method as implemented in the R package [54] with ‘~treatment*sc_time’ as the design formula. The normalized count matrix was then transformed using a variable-stabilizing transformation (vst), followed by principal coordinate analysis (PCoA) on the Euclidean distances. A db-RDA was then performed using the retained PCoA factors as the response matrix and the variables ‘time’, ‘batch’ and ‘viral load’ as the explanatory matrix. A total of six PCoA axes were retained based on the Gower dissimilarity index, explaining 66.86% of the total variance. Three partial db-RDAs and permutation tests for constrained ordination (*N*=1,000 permutations) were performed to validate the effect of each factor while controlling for others.

All R analyses described in this section can be found in script 05 R_script_1_sequencing_analysis_raw on GitLab.

### OsHV-1 gene expression analysis

To access OsHV-1 RNAseq coverage, the .sam files produced by STAR were processed using Samtools v.1.16.1 [55] with parameters samtools view -f 128 and -F 16, -f 64 and -F 32, -f 144, -f 96, to select forward and reverse aligned reads and samtools merge -f to merge all forward and all reverse aligned reads. Then bed coverage files (i.e. coverage per base on the OsHV-1 genome) were generated using Bedtools v.2.30.0 [56] with bedtools genomecov with standard parameters (Script 03-Generate_bed_files in GitLab). Reads per million normalization was applied to all bed coverage files by dividing the coverage at each base by the total number of reads in the library and multiplying the result by 1×10^6^.

For OsHV-1 genome coverage evaluation over time and across batches, replicate data were averaged for each base of the genome. Antisense and sense reads of the OsHV-1 ORFs were identified using the orientation of the alignments and the OsHV-1 genome annotation with a custom script. The ratio of antisense to sense reads was also calculated for each position and each time point.

To compare viral ORF expression over time and across batches, the forward count matrix of all genes (i.e. *M. gigas* and viral ORFs) was normalized using DESeq2 version 1.46.0 [54], with a design formula that included ‘time’, ‘batch’ and their interaction ‘time+batch+time:batch’. Viral ORFs were extracted from the normalized count matrix and further normalized by the length of the OsHV-1 ORFs per kilobase. We first evaluate the expression of new and modified ORFs, considering ORFs as expressed when the normalized count was greater than 1 reads per million per kilobases (RPKM).

To analyse viral gene expression profiles over time, we applied a vst on the DESeq2 object containing all virus and host genes and extracted ORF data from this matrix. We then computed the Euclidean distances for average-linkage hierarchical clustering and removed ORFs that were never expressed at any time points from the dataset and cut the resulting dendrogram into three clusters. Differences in expression dynamics between host batches were assessed using Wilcoxon rank-sum tests [57] at each time point within clusters, and adjusted *P*-values were calculated using the Benjamini–Hochberg procedure [58].

Finally, the expression of ORFs in LS and HS batches was compared by performing statistical tests at each time point on the DESeq2 object. An ORF was considered differentially expressed if the absolute value of its log_2_ fold-change was greater than 1 and if its adjusted *P*-value (Benjamini–Hochberg method) was less than 0.05. A negative log_2_ fold-change indicated that the ORF was overexpressed in the LS batch, whereas a positive log_2_ fold-change indicated overexpression in the HS batch.

All R analyses described in this section can be found in script 06 R_script_2_OsHV-1_ORFs_analysis_raw on GitLab.

### *M. gigas* gene expression analysis

To analyse Pacific oyster gene expression, genes were extracted from the previous vst matrix. Low-variance genes (i.e. genes with variance less than 0.05) were removed from the dataset.

First, to analyse the response of Pacific oyster genes to the viral infection according to the time for each batch, we performed a weighted correlation network analysis (WGCNA) using the R package WGCNA [59] on all infected libraries. To do so, we created a design matrix including ‘LS.time’, ‘HS.time’, ‘cluster 1’, ‘cluster 2’ and ‘cluster 3’ corresponding to the eigenvalues of each of the three viral clusters per library and ‘batch’. The soft power threshold was set at 10, using the scale-free topology criterion, achieving a model fit of 0.78 and an average connectivity (k) of around 275. Gene modules were identified using the cutreeDynamic function, with a minimum of 50 genes per module.

Then, to analyse the response of oyster genes to the time for each batch, we performed a WGCNA analysis using the R package WGCNA version 1.73 [59] on all control libraries. To do so, we created a design matrix including ‘time’ and ‘batch’. The soft power threshold was set at 9, using the scale-free topology criterion, achieving a model fit of 0.76 and an average connectivity of around 379. Gene modules were identified using the cutreeDynamic function, with a minimum of 50 genes per module.

For each module of each WGCNA analysis, the module membership was defined, and the correlation between the module eigengene value and condition traits was assessed. Modules that showed a significant correlation (i.e. *P*<0.005) with the interested traits were retained, and genes belonging to these modules were extracted.

To perform functional enrichment analysis, Gene Ontology (GO) terms were assigned to Pacific oyster genes. Functional annotation of the oyster genome was performed using EggNOG-mapper v2 [50] for orthology-based inference and blastp [60] searches against the curated UniProt-SwissProt database (released January 2023 [61]) for high-confidence protein annotations (threshold *e*-value <10e-5). Finally, a single file combining all GO annotations was generated with the nrifyGOtabl.pl script of the GO_MWU tool [62] (Script 04-GO_annotations in GitLab).

Custom scripts were then used to extract the best hits, retrieve GO terms from the blast and EggNoG-mapper outputs. Functional enrichment analysis was performed on these genes using a rank-based GO approach with adaptive clustering, applying a Mann–Whitney *U* test for each independent module using the GO_MWU R package [62] with the go.obo database downloaded in January 2025. REviGO was used to reduce redundancy in GO terms [63]. To visualize functional enrichment, the negative logarithm of the adjusted *P*-value and the ratio of significant genes to total genes were calculated for each GO.

All R analyses described in this section can be found in script 07 R_script_3oyster_genes_analysis_raw on GitLab.

## Results

### Sequencing summary

#### Data quality and outlier removal

Of the 94 libraries (i.e. 46 for the LS batch and 48 for the HS batch), the total number of reads per library ranged from 9 to 20 million, with an average of 13 million reads. Alignments to the Pacific oyster and virus genomes showed that 88–94% of the reads of all libraries aligned to the Pacific oyster genome (average: 93%). For the infected condition, 0.00022–0.31651% of the reads from the LS batch libraries aligned to the OsHV-1 genome (average: 0.083%), whereas 0.00023–2.69% of the reads from the HS batch libraries aligned to the OsHV-1 genome (average: 0.39%). Finally, between 5.95 and 11.97% of the reads did not align to either *M. gigas* or OsHV-1 genomes in the LS batch (average: 7.02%), and between 6.06 and 8.96% of the reads did not align to either *M. gigas* or OsHV-1 genomes in the HS batch (average: 7.0%) (Fig. S1).

By visualizing the 5,000 most variable genes in a heatmap, we identified 5 outlier libraries that were subsequently removed from the analysis: in the LS batch, the first replicates of the non-infected condition at 1 and 4 hpi, as well as the third replicate at 18 hpi; in the HS batch, the second replicate of the non-infected condition at 18 hpi and the first replicate of the infected condition at 18 hpi (Fig. S2). The transcriptomic profiles of these libraries deviated markedly from those of their respective replicates. Although sequencing quality metrics were comparable to those of the other libraries, these discrepancies likely reflect variability introduced during sampling, RNA extraction or library preparation.

#### Oyster batch and time post-infection drive the variability of the whole expression

**Fig. 2.**
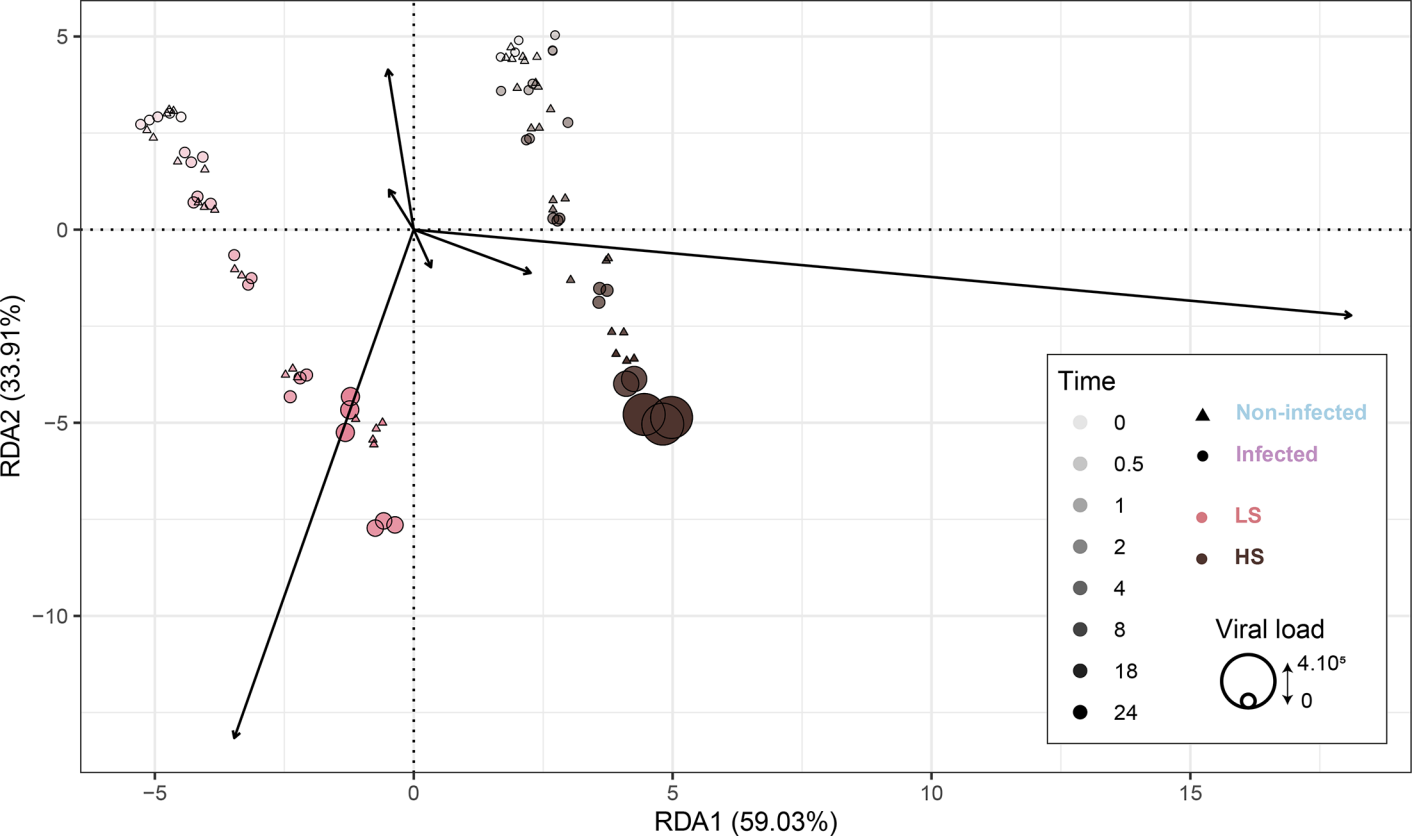
RDA plot. The *X*-axis represents the first RDA component, which explains 59.03% of the variance, and the *Y*-axis represents the second RDA component, which explains 33.91%. The dot shape indicates treatment, with triangles for non-infected libraries and circles for infected libraries. Dot size corresponds to the viral load (i.e. OsHV-1 copy number per ng of extracted DNA). Colour corresponds to oyster batch, with pink for the LS batch and dark brown for the HS batch. The shade of the dot indicates the time after infection: light pink or brown dots correspond to early time points, while dark pink or brown dots correspond to later time points.

The redundancy analysis (RDA) plot illustrates the variance in gene expression between oyster batches under two treatments: non-infected and infected, across time points (Fig. 2). The first axis of the RDA (RDA1), accounting for 58.1% of the variance, shows a clear separation between the LS and the HS batch libraries. The second principal axis (RDA2), explaining 34.58% of the variance, further differentiates samples over time. These results suggest that both factors, ‘batches’ and ‘time’, significantly influence the gene expression patterns. This is verified by the adjusted *R*^2^ of the partial RDA showing that 14.40% of the variance is explained by the ‘time’, 15.73% by the ‘batches’ and 8.91% by the ‘viral load’.

#### Viral genomes, copy number and viral transcription levels are higher in the HS batch

**Fig. 3.**
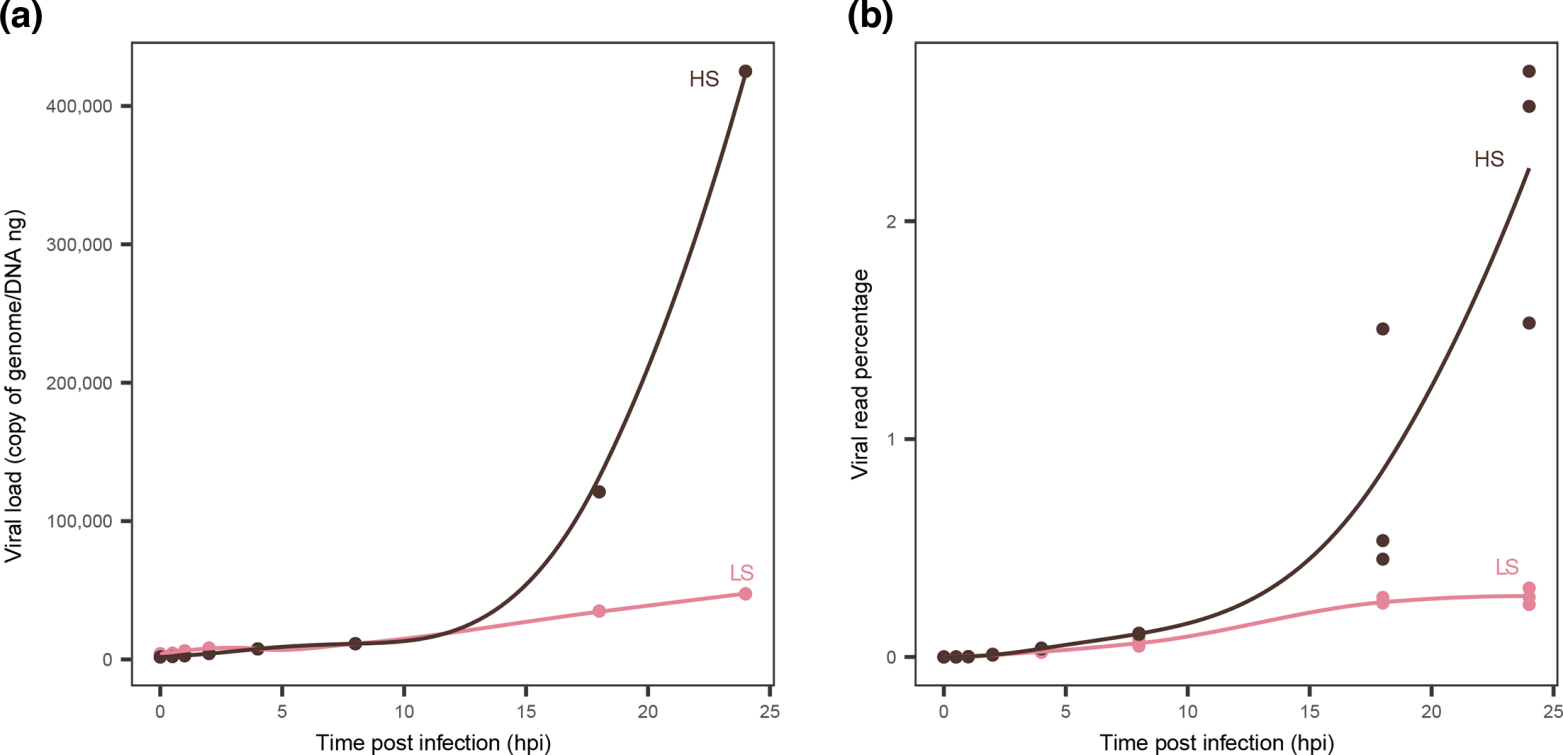
Viral DNA and RNA quantity over time. (a) Viral load in copies of genome per ng of DNA over time. Each point represents a sample coloured by the haemocyte batch they belong to (pink for the LS batch and dark brown for the HS batch). The *Y*-axis represents viral DNA copies per ng of DNA, while the *X*-axis corresponds to time in hours post-infection. (b) Percentage of viral reads over time. Each point represents a cDNA library, coloured by the batch they belong to (pink for the LS batch and dark brown for the HS batch). The *Y*-axis represents the percentage of OsHV-1 reads, while the *X*-axis represents the time in hours post-infection.

Our qPCR results showed that the OsHV-1 copy number in DNA extracted from haemocytes of LS oysters increased slowly over time, reaching a maximum of 4.7×10^4^ copies per ng of DNA at 24 hpi. In contrast, haemocytes from HS oysters showed a rapid increase, reaching 4.3×10^5^ copies per ng of DNA (Fig. 3a).

A similar pattern appears in the percentage of viral reads from the RNAseq between the LS and the HS batches. For the LS batch, the percentage increased slowly to 0.3% at 24 hpi, whereas it increased quickly to a maximum of 2.6% at 24 hpi for the HS batch (Fig. 3b).

### OsHV-1 gene expression

#### Updated OsHV-1 gene prediction reveals new and modified ORFs

Our updated ORF prediction identified 144 ORFs, of which 5 were modified compared to the OsHV-1 µVar A genome (GenBank: KY242785) and 17 were newly predicted (Table S1). The five modified ORFs include three that are longer than previously described (ORFS17, ORFS32.3 and ORFS115), one that is shorter (ORFS114) and one that results from the fusion of ORFIN1 and ORFIN2 (ORFSIN1.2) (Table S1). Of the new ORFs, three (ORFS4.2, ORFS105.3 and ORFS116.1) exhibited expression levels below 1 RPKM in both batches at all time points. ORFS32.1 was also lowly expressed in the LS batch, but exceeded 1 RPKM at 18 hpi and 24 hpi in the HS batch. The functions of these four ORFs are unknown, but predictions show they may be addressed to the endoplasmic reticulum (ORFS32.1) or in the nucleus (ORFS4.2, ORFS105.3 and ORF116.1) (Table S1).

The remaining 13 newly predicted ORFs and the 5 modified ORFs were expressed above 1 RPKM in both batches. None of them has known functions, but they are predicted to localize in the cytoplasm or nucleus (ORFS32.2, ORFS33.1, ORFS119.2, ORFS50, ORFS69.1, ORFS72.1, ORFS105.4, ORFS4.1, ORFS43.3, ORFS115.2 and ORFS118.1), the extracellular region (ORFS121.1) and the membrane (ORFS62).

Of the five modified ORFs, only ORFS115 had a known function (an origin-binding replication protein [15]. The four others showed predicted features, including a coiled-coil domain in ORFS17, a disordered region in ORFS32.3 and a signal peptide in ORFSIN1.2. Their predicted localizations were nuclear (ORFS17 and ORFS115), cytoplasmic (ORFS114), membrane (ORFS32.3) and extracellular (ORFSIN1.2) (Table S1).

#### OsHV-1 gene expression dynamics highlight differences between the two Pacific oyster batches

To investigate OsHV-1 gene expression dynamics, viral genome coverage and normalized counts were analysed over time by focusing on the 46 OsHV-1 infected libraries.

**Fig. 4.**
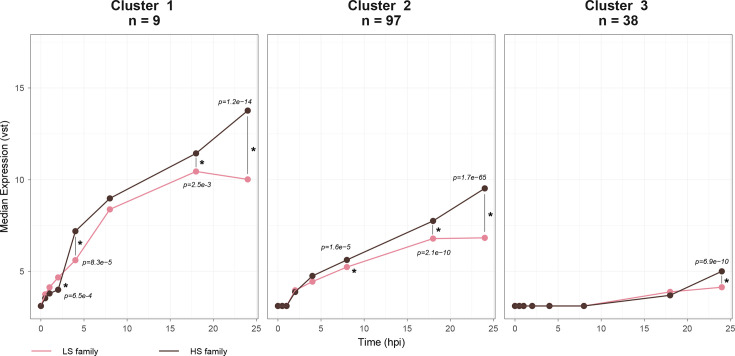
Clustering of viral genes based on their expression. Each panel shows one of the three clusters. The *Y*-axis shows the median expression, transformed using a vst, and the *X*-axis shows time in hours post-infection. Pink lines and dots represent the LS batch, and dark brown lines and dots represent the HS batch.

Three distinct clusters of viral genes were identified based on their expression profiles (Table S1). Cluster 1 includes 9 genes, cluster 2 includes 97 genes and cluster 3 includes 38 genes (Fig. 4). Cluster 1 genes are expressed immediately after infection with a median onset after 0 hpi. This cluster includes ORF27 coding dUTPase-like, predicted to localize in the cytoplasm; ORF45 and ORF107 coding proteins with disordered regions, predicted to localize in the nucleus or cytoplasm; ORF80 coding a transmembrane glycoprotein with a signal peptide, predicted to localize in the Golgi apparatus; ORF88 and ORF111 coding transmembrane glycoproteins; and ORF82, ORF104 and ORF122 coding proteins of unknown function, predicted to localize in the nucleus (Fig. 4, Table S1).

Cluster 2, the largest group, is characterized by early expression, with a median onset after 1 hpi. This cluster includes several key functional genes, such as ORF75, which encodes a dUTPase-like protein, four genes involved in the inhibition of apoptosis (ORF42, ORF87, ORF99 and ORF106) and eight out of the nine known genes involved in the DNA replication and coding for ribonucleotide reductase subunits (ORF20 and ORF51), DNA primases (ORF7, ORF49 and ORF66), DNA helicase (ORF67), DNA polymerase (ORF100) and a mitochondrion-localized P-loop containing nucleoside triphosphate hydrolase (ORF44), all predicted to localize in the cytoplasm and/or nucleus (Fig. 4, Table S1).

Cluster 3 genes are predominantly expressed at later stages of infection, with a median onset after 8 hpi in both batches. Notably, this cluster includes 15 modified or newly annotated ORFs, as well as ORF109, which codes a putative DNA packaging protein (Fig. 4, Table S1).

The expression dynamics of these clusters differed markedly between host batches (Fig. 4). In the LS batch, gene expression rose until 18 hpi before declining or stabilizing at 24 hpi, whereas in the HS batch, it continued to increase throughout the 24 hpi period, resulting in overall lower viral expression levels in the LS batch. These contrasting trajectories were supported by statistical analyses: in cluster 1, differences between batches emerged as early as 2 hpi and remained highly significant thereafter (*P*<1×10^−14^ at 24 hpi); in cluster 2, divergence became significant from 8 hpi onward, with very big differences at 18 hpi and 24 hpi (*P*<1×10^−10^ and *P*<1×10^−65^, respectively); and in cluster 3, batches remained comparable until a pronounced difference appeared at 24 hpi (*P*<1×10^−10^) (Fig. 4).

**Fig. 5.**
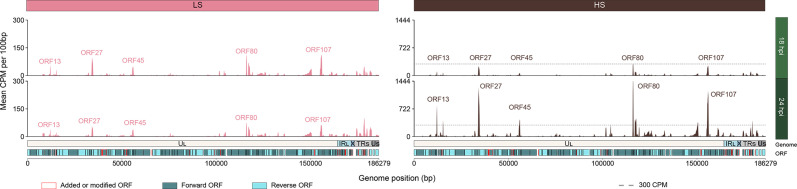
OsHV-1 whole-genome expression over time and oyster batches. The mean counts per million (CPM) per 100 bp windows are represented on the *Y*-axis for the 18 hpi library on the top and the 24 hpi on the middle. The plot of the LS batch data, on the right, is coloured in pink, whereas the plot of the HS batch data, on the left, is coloured in dark brown. The *X*-axis corresponds to the OsHV-1 genome position in base pairs. Representations of the OsHV-1 genomic regions (U_L_ in white, IR_L_ in light blue, X in dark blue, IR_S_ in light grey and U_S_ in dark grey) and the ORF location on the genome are shown above the *X*-axis. ORFs coloured in dark blue are forward-directed ORFs, and ORFs coloured in light blue correspond to reverse-directed ORFs. Finally, ORFs surrounded by red colour correspond to new or modified ORFs compared to the OsHV-1 µVar A genome. The *Y*-axis of the LS batch ranged from 0 to 300 CPM, while it ranged from 0 to 1444 CPM for the HS batch. Dashed lines on plots of the HS batch represent the maximum of CPM in the LS batch.

By the end of infection, five ORFs (ORF13, ORF27, ORF45, ORF80 and ORF107) had clearly higher levels of expression compared to the other ORFs in both batches (Figs 5 and S3). In the LS batch, these genes reached their highest expression level at 18 hpi, before declining at 24 hpi. By contrast, in the HS batch, their expression increased strongly between 18 and 24 hpi, showing a four- to ninefold increase and reaching levels up to 10 times higher than in the LS batch at 24 hpi (Fig. 5). All five genes belong to cluster 1, except for ORF13 (protein containing a signal peptide domain, predicted to be extracellular), which belongs to cluster 2 (Table S1).

#### OsHV-1 antisense/sense gene expression ratios differ between the two Pacific oyster batches

**Fig. 6.**
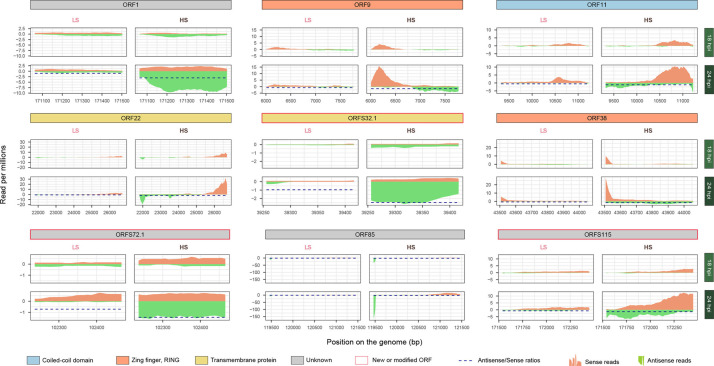
Sense and antisense coverage for selected OsHV-1 ORFs. Each panel represents a different ORF. The colour of the header represents the protein’s predicted function or domain of each ORF. Red surrounding of the header means that the ORFs were new or modified compared to the OsHV-1 µVar A genome. For each panel, the two graphs on the right represent the data for the LS batch at 18 and 24 hpi, whereas the two graphs on the left represent the data for the HS batch at 18 and 24 hpi. The *Y*-axis of each graph represents the read count per million, and the *X*-axis represents the position of the ORF on the OsHV-1 genome. Orange coverage on the top of each graph represents the sense coverage, while the green one represents the antisense coverage.

To investigate the putative regulation of gene expression by antisense transcripts, we analysed the antisense/sense expression ratios of viral genes over time. Overall antisense/sense ratios remained stable across time (Fig. S5). However, at later infection stages, some ORFs showed more sense than antisense reads in the LS batch, whereas the same ORFs showed more antisense than sense reads in the HS batch. This is the case for ORF85, which had a ratio of 0.87 in the LS batch and a ratio of 1.51 in the HS batch at 18 and 24 hpi. Similarly, ORF1, ORF9, ORF11, ORF22, ORFS32.1, ORF38, ORFS72.1 and ORFS115 showed a ratio from 0.59 to 0.97 in the LS batch and a ratio from 1.18 to 3.05 in the HS batch at 24 hpi (Figs 6, S3). With the exception of ORFS115, which has been identified as an origin-binding replication protein [15], none of these ORFs has a predicted function. However, ORF1, ORF9, ORF85, ORFS72.1, ORF22, ORFS115 and ORF11 are predicted to localize to the nucleus, ORF38 to the cytoplasm and ORFS32.1 to the endoplasmic reticulum. ORF9 and ORF38 contain a zinc/RING finger domain, whereas ORFS32.1, ORF22 and ORF38 have a transmembrane domain. ORF22 and ORF11 contain a consensus disorder region, and ORF11 also contains a coil domain (Table S1).

Notably, none of the ORFs showed the reverse pattern of having more antisense than sense reads in the LS batch compared to the HS batch.

#### OsHV-1 differential expression analysis reveals differences in infection dynamics between the two Pacific oyster batches

**Fig. 7.**
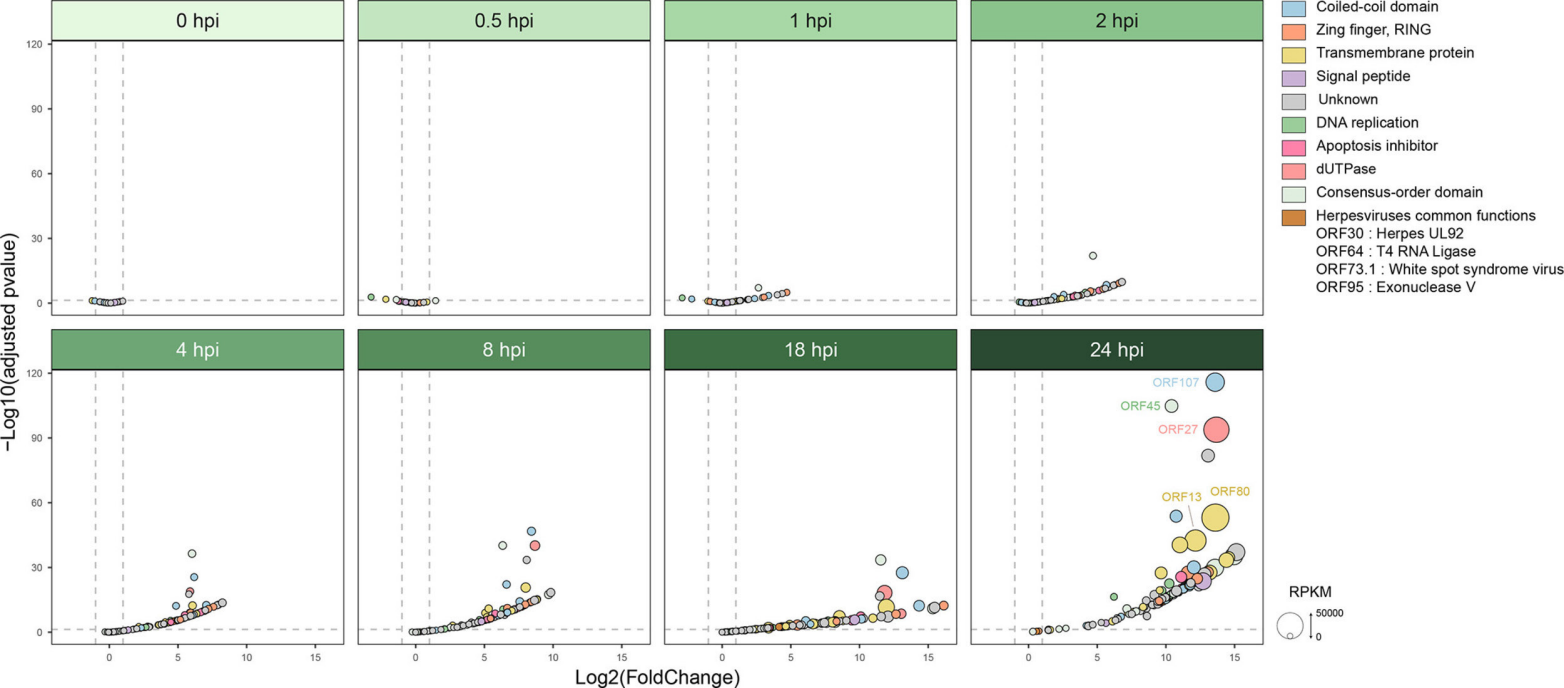
Volcano plot of differentially expressed viral genes. Differential expression analysis was performed using DESeq2 to compare viral gene expression between the HS and LS oyster batches. The *X*-axis shows the log2 fold-change (Log2FC), and the *Y*-axis shows the negative log10 of the adjusted *P*-value (-Log10(padj)). The vertical dashed lines indicate a Log2FC threshold of ±1, and the horizontal dashed line marks the significance threshold for the adjusted *P*-value padj=0.05. Each point represents a viral gene, coloured according to its function or domain. Point size reflects normalized expression in RPKM. Genes to the left of the thresholds are overexpressed in the LS batch, while those to the right are overexpressed in the HS batch.

Statistical analyses were conducted to compare normalized expression levels between both batches at each time point. During the early stage of infection (before 1 hpi), four genes were significantly overexpressed in the LS batch: ORF49, ORF74 and ORF122 at 0.5 hpi and ORF49 and ORF90 at 1 hpi (Fig. 7, Table S1).

Then from 2 to 24 hpi, all genes were overexpressed in the HS batch compared to the LS one (Fig. 7). Notably, genes coding dUTPase-like, ORF75 and ORF27, started to be overexpressed in the HS batch at 1 and 4 hpi, respectively. Moreover, genes involved in the apoptosis inhibition (ORF42, ORF87, ORF99 and ORF106) started to be overexpressed in the HS batch at 2 hpi. Finally, for the genes involved in the DNA replication, ORF20, ORF67 and ORF44 started to be overexpressed in the HS batch at 2 hpi, ORF7, ORF66 and ORF100 at 4 hpi, ORF109 at 8 hpi and 18 hpi and ORF49 at 24 hpi (Fig. 7, Table S1).

### Oyster gene expression

#### Haemocytes of both Pacific oyster batches respond to viral infection

To investigate Pacific oyster gene expression in response to OsHV-1 infection, we conducted a WGCNA analysis on infected libraries from both batches. This approach identifies 59 gene modules, 4 of which (dark turquoise, grey60, honeydew and red) were significantly correlated with time and viral gene cluster expression (Fig. S5).

**Fig. 8.**
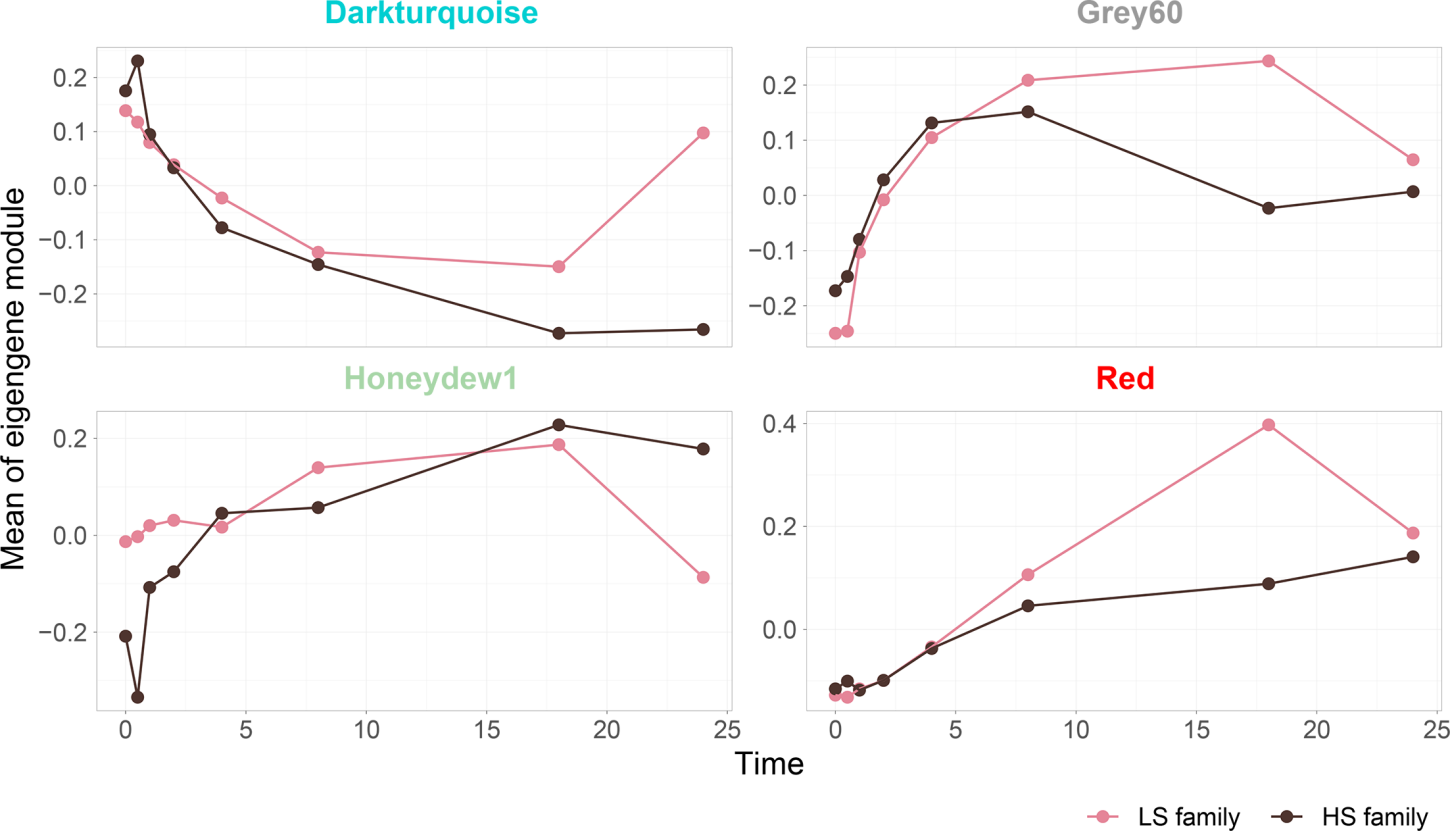
Eigengene value over time according to oyster immune-related WGCNA modules. Each panel shows the eigengene profile of an immune-related module identified through WGCNA analysis. The *Y*-axis shows the mean eigengene value for each module, and the *X*-axis shows time in hpi. Pink lines and dots represent the LS batch, while dark brown lines and dots represent the HS batch.

The dark turquoise module, containing 5,057 genes, exhibited a robust negative correlation with viral clusters and time in the HS batch and a moderate negative correlation with time in the LS batch (Fig. S5). Gene Ontology (GO) enrichment analysis revealed that this module is primarily associated with cell mobility and enzymatic signalling, encompassing processes such as locomotion, cell adhesion and morphogenesis (Table S2). The value of the eigengene steadily declined over time in the HS batch, whereas in the LS one, it initially declined, reaching a minimum at 18 hpi, before rising by 24 hpi (Fig. 8).

The grey60 module, containing 1,340 genes, was positively correlated with both viral clusters and time in the LS batch, and, to a lesser extent, with time in the HS one (Fig. S5). Go terms associated with this module were related to vesicular trafficking and Golgi apparatus organization (Table S2). For the HS batch, the eigengene value increased until 8 hpi, then decreased through 18 hpi and increased again towards 24 hpi, whereas in the LS, it increased until 18 hpi, before decreasing by 24 hpi (Fig. 8).

The honeydew1 module, containing 514 genes, showed a strong positive correlation with viral clusters and time in the HS batch and a moderate positive correlation with time in the LS one (Fig. S4). Enriched GO terms suggested involvement in metabolic regulation, protein degradation, mitochondrial function and immune-related signalling pathways (Table S2). For the HS batch, the eigengene value increased continuously, whereas in the LS batch, it rose until 18 hpi and then decreased by 24 hpi (Fig. 8).

Finally, the red module, containing 1,668 genes, exhibited the strongest positive correlation with both viral clusters and time in both batches (Fig. S5). GO enrichment analysis revealed a dominant involvement with immune responses and the regulation of cellular stress (Table S2). The eigengene value increased slightly over time in the HS batch, whereas, in contrast, it increased rapidly to 18 hpi in the LS batch, before declining by 24 hpi (Fig. 8).

#### Expression of most Pacific oyster genes drifts over the course of the experiment

Finally, to evaluate the effect of the experiment duration on Pacific oyster gene expression, we performed a WGCNA analysis on all control libraries. We identified 57 modules, 8 of which were significantly correlated with the time. The black, dark violet, dark turquoise, yellow and yellow-green modules, containing 4,647 genes, showed a strong positive correlation with time, whereas the magenta, grey60 and sky blue modules, containing 4,813 genes, exhibited a strong negative correlation with time (Fig. S6).

GO enrichment analysis revealed that the negatively correlated modules are enriched for terms associated with the downregulation of key signalling pathways and the modulation of cellular architecture and chromatin dynamics. Conversely, the positively correlated modules were enriched for GO terms supporting mitochondrial bioenergetics and macromolecular assembly, including processes such as energy production, protein folding and RNA processing (Table S3).

Among those 9,460 genes, 5,853 were shared with the immune-related modules identified previously (Table S4).

## Discussion

The study aimed to improve our understanding of the OsHV-1 lytic phase in haemocytes from two Pacific oyster batches that exhibit different levels of susceptibility to OsHV-1 infection. First, we found that both viral DNA and RNA were detected in both batches, indicating that OsHV-1 can infect and replicate in haemocytes. A similar pattern was observed in *M. gigas* tissue during OsHV-1 infection, with higher levels of viral DNA and RNA for HS Pacific oysters [23,40].

Then, to better understand both the dynamics of OsHV-1 replication and the immune response of haemocytes over time for both batches with contrasting responses to OsHV-1 infection, we used time-series dual RNA sequencing.

The expression of 144 OsHV-1 genes was monitored over a 24-h period. Among them, 5 corresponded to modified ORFs from previous OsHV-1 annotation [15], and 17 were newly predicted ORFs. Nineteen of these 22 genes were expressed during the course of infection, suggesting active transcription and a potential role in the lytic phase. However, three genes (ORFS4.2, ORFS105.3 and ORFS116.1) were not expressed more than 1 RPKM, suggesting that these genes were not expressed under these experimental conditions or because of incorrect ORF prediction.

To decipher the timing of the whole OsHV-1 gene expression, we first studied the viral infection over time. Clustering the viral genes into three groups, based on their expression, revealed that some viral genes were already expressed at 0 hpi in both batches. Although this time point is slightly delayed due to the centrifugation step between the collection of haemocytes and RNA extraction, the detected gene expression suggests that OsHV-1 replication begins rapidly after infection. This observation indicates a faster onset of viral expression than previously described in haemocytes (1 hpi) or in the mantle (2 hpi) [23,34].

During the lytic phase of vertebrate herpesviruses, in which this phase has been studied, gene expression follows a sequential pattern. Immediate-early genes are expressed shortly after the onset of infection and transcribed without the need for prior viral protein synthesis, then early genes are expressed at an intermediate stage and are transcribed with the help of immediate-early proteins, and finally, late genes are expressed at the end of infection once the replication of the viral genome starts [2,3, 64]. Although these three categories may overlap in expression [2,65], immediate-early and early gene expression typically declines during the infection [64,66,70].

We identified 9 genes that are expressed immediately after the onset of infection (i.e. after 0 hpi), 97 that are expressed early (i.e. after 1 hpi) and 38 that are expressed more lately (i.e. after 8 hpi). This represents an improvement on a previous study that analysed the transcriptome of OsHV-1 using qPCR to target 39 ORFs, which did not allow classification into immediate-early, early and late genes [23,36]. This is likely related to the restricted selection of target genes, which were predominantly confined to a single functional class (i.e. 31/39 of their selected genes belonged to the early cluster in our study). In contrast, this whole-genome transcriptomic analysis of OsHV-1 provides a more precise evaluation of all viral ORFs, eliminating the bias introduced by targeting only a subset of genes via qPCR.

Moreover, in contrast to what has been shown for vertebrate herpesviruses, OsHV-1 gene expression increases over time and is cumulative. While this pattern could be specific to OsHV-1, it may be influenced by the presence of multiple haemocyte cell types in the primary culture that respond differently to viral infection, masking the sequential expression profile of early genes [71,73]. For example, *M. gigas* granulocytes are more immunocompetent to viral infection than hyalinocytes, which could lead to a delayed response to infection for this type of haemocyte within the overall cell population [74]. Single-cell transcriptomics could clarify the specific roles of haemocyte subtypes in the antiviral response and reveal expression dynamics masked in bulk analyses.

However, the expression of some OsHV-1 genes harbours similarities with vertebrate herpesviruses. In particular, genes coding for a dUTPase-like protein, apoptosis inhibitor and genes involved in the DNA replication belong to clusters that are characterized by an expression early after the onset of infection in Pacific oyster haemocytes, similarly to what has been observed in the mantle of high-susceptible Pacific oyster but also in human herpesviruses [3,6, 36, 75,77]. Interestingly, five genes harbour very high expression levels at the end of the infection, with an even more pronounced trend in the HS batch. Unfortunately, except for ORF27, which codes for a dUTPase-like protein, none of the four remaining genes has a functional annotation. However, these genes contain predicted domains and predicted localization that suggest that they could be involved in the final assembly of the virion. For example, ORF80 possesses a transmembrane domain and is predicted to be localized in the Golgi apparatus, which is known to be involved in the acquisition of the final envelope and tegument of the virion in herpesviruses, and ORF13 contains a signal-peptide and is predicted to be located in the extracellular region and could be involved in the late stage of the progeny virion assembly [2,3, 78]. Moreover, ORF27 was identified as highly expressed in the mantle of other high- and low-susceptible Pacific oysters [23]. Proteins coded by ORF107, ORF27 and ORF45 have also been identified as highly present in other high-susceptible Pacific oysters infected by OsHV-1 [79], and ORF107, which is suggested to code for a capsid maturation protease, was among the most abundant genes expressed in OsHV-1 isolate ZK0118 infecting clam haemolymph [80]. These findings suggest that these highly expressed genes may be involved in the assembly of the tegument and envelope of progeny virions released from infected cells. This is further reinforced by the observation that genes associated with virion assembly are among the most highly expressed in herpesviruses [3,68, 81]. However, in other herpesviruses, genes involved in the assembly of the virion are generally classified as late genes [9,66]. In this study, four of these five genes were grouped into cluster 1, characterized by expression that occurs immediately after infection onset.

Stranded sequencing allowed us to investigate the potential role of an antisense transcript in the regulation of viral expression. The analysis of the antisense vs. sense transcripts ratio shows that some genes harbour more antisense transcripts in the HS batch than in the LS one at later stages of OsHV-1 infection. Antisense transcripts can interact with mRNAs and may play a key role in post-transcriptional regulation. They may be involved in mRNA stabilization, regulation of translation, maturation, transport or localization and modification of proteins [82,83]. Antisense transcripts of lytic herpesvirus genes have been reported, although their function remains unclear. For example, ~50% of the viral genome of HSV-1 produces abundant symmetric transcripts at late stages of infection [84,86], while several antisense transcripts have been found overlapping ORFs in HCMV [87]. Here, the antisense versus sense ratios suggest that some OsHV-1 genes produce antisense transcripts. The observed differences between oyster batches may reflect a regulatory mechanism in the HS batch aimed at slowing viral replication to prevent rapid cell destruction, giving the virus a longer time frame to replicate before cell death.

To further understand the interactions between the virus and its host, we next investigated the transcriptional response of the Pacific oyster to the OsHV-1 infection. By comparing the gene expression in haemocytes from both Pacific oyster batches of infected libraries, we aimed to identify host pathways that are modulated during OsHV-1 infection and to determine how these might contribute to the contrasting levels of susceptibility to viral infection. To this end, we characterized co-expression modules of host gene expression correlated with OsHV-1 infection for each contrasted oyster batch, identifying four modules that highlighted distinct patterns depending on the batch. The first module contained genes associated with cell mobility, adhesion and morphogenesis, the second module included genes involved in vesicular trafficking and Golgi apparatus function and the final two were enriched in genes related to the immune response and the regulation of cellular stress.

On the one hand, in the LS batch, the expression of genes involved in cell mobility, adhesion and morphogenesis decreased until 18 hpi before increasing sharply. In contrast, genes associated with vesicular trafficking, the Golgi apparatus, the immune response and cellular stress regulation showed a marked increase until 18 hpi, followed by a decline. Furthermore, expression levels of the three viral gene clusters increased in the LS batch until 18 hpi and then stabilized or decreased.

In the HS batch, the expression of genes involved in cell mobility, adhesion and morphogenesis decreased until the end of the experiment. In contrast, genes associated with vesicular trafficking, the Golgi apparatus, the immune response and cellular stress regulation increased until the end of the experiment. Moreover, expression levels of the three viral gene clusters increased in the HS batch until the end of the experiment.

Taken together, these findings suggest that haemocytes from LS oysters activate a more effective and regulated immune response than those from HS oysters, thereby limiting viral replication at 18 hpi. This response appears to be accompanied by a reduction in vesicular trafficking and Golgi activity, while processes related to cellular motility, adhesion and morphogenesis are reactivated. In contrast, the immune response of haemocytes from the HS oysters may be too slow to control the infection as OsHV-1 genes are already highly expressed and viral particles are widely distributed within the haemocytes. These patterns have been previously observed with oysters from another LS batch, which responded earlier to the OsHV-1 infection (before 24 hpi), while oysters from another HS batch responded later (after 24 hpi) [32].

We next examined how haemocyte gene expression varied throughout the experiment by comparing the control libraries from the low- and high-susceptibility batches. We identified five gene modules positively correlated with the time and three negatively correlated. GO enrichment analysis revealed that haemocytes initially mobilize resources to survive in culture, but gradually lose regulatory balance, resulting in progressive functional decline. As no viral material was detected in these control libraries, it is likely that the duration of the experiment affected the survival potential of haemocytes. Furthermore, 68% of the genes in the immune-related modules were also present in the time-related modules, confirming our redundancy analysis, where the time accounts for 14.40% of the explained variance, whereas OsHV-1 infection explains only 8.91%. Taken together, these results suggest that haemocytes are more strongly affected by culture-related stress than by OsHV-1 infection itself. This finding indicates that the current primary haemocyte culture approach may not be suitable for long-term time-series experiments. The observed limitation likely stems from the presence of antibiotics in the culture medium and from the inherently suboptimal conditions associated with prolonged *in vitro* maintenance of oyster haemocytes. Although no comparable data are currently available for oyster primary cultures, studies in human cell systems have shown that antibiotics can induce cellular stress responses and consequently alter host transcriptomic profiles [88,89]. More recently, a plasma-based culture medium has been developed, enabling primary haemocyte cultures to remain viable and functional for up to 21 days [90,91]. To better assess the influence of culture conditions and antibiotics on host gene expression during infection, reproducing the present experiment using this new plasma-based system would be highly informative. Furthermore, the lack of microscopic monitoring of haemocyte primary cultures in the current study limits the interpretation of the transcriptomic data and prevents any evaluation of haemocyte morphology or behaviour over the course of the experiment.

Finally, for the first time, we conducted a time-series dual RNA sequencing on viral-infected haemocytes from two oyster batches harbouring different levels of susceptibility to OsHV-1 infection. This study deciphers the sequential pattern of viral gene expression, providing new insights into the replication dynamics of OsHV-1 in Pacific oyster haemocytes. It also reveals how the immune response to infection varies between batches, highlighting the role of host genetics in shaping the outcome of infection. However, our results also suggest that haemocytes maintained in artificial seawater may exhibit time-dependent stress responses that may confound infection studies. This highlights the importance of experimental conditions and suggests that future time-series analyses should take into consideration cellular stress unrelated to viral infection. However, while our work focused exclusively on haemocytes, which play a crucial role in oyster immunity [39], extending such analyses to other oyster tissues would be highly valuable to assess potential differences in OsHV-1 infection dynamics and to determine whether these tissues exhibit similar or distinct responses to the virus.

## Abbreviations

db-RDA: distance-based redundancy analysis
GO: gene ontology
hpi: hours post-infection
HS: high-susceptible
HSV-1: herpes simplex virus 1
LS: low-susceptible
OsHV-1: Ostreid herpesvirus 1
PCoA: principal coordinate analysis
qPCR: quantitative PCR
RDA: redundancy analysis
RPKM: reads per million per kilobases
vst: variable-stabilizing transformation
WGCNA: weighted correlation network analysis

## References

[1] Silva JM, Pratas D, Caetano T, Matos S (2022). The complexity landscape of viral genomes. Gigascience.

[2] Everett RD, Diefenbach RJ, Fraefel C (2014). Herpes Simplex Virus: Methods and Protocols.

[3] Knipe DM, Howley PM, Cohen JI, Griffin DE, Lamb RA (2013). Fields Virology. 6th Edn.

[4] Jones PC, Roizman B (1979). Regulation of herpesvirus macromolecular synthesis. J Virol.

[5] Packard JE, Dembowski JA (2021). HSV-1 DNA replication-coordinated regulation by viral and cellular factors. Viruses.

[6] Wofford AS, McCusker I, Green JC, Vensko TA, Pellett PE, Kielian M, Mettenleiter TC, Roossinck MJ Advances in Virus Research.

[7] Davison AJ (2002). Evolution of the herpesviruses. Vet Microbiol.

[8] Davison AJ (2010). Herpesvirus systematics. Vet Microbiol.

[9] Dotto-Maurel A, Arzul I, Morga B, Chevignon G (2025). Herpesviruses: overview of systematics, genomic complexity and life cycle. Virol J.

[10] Davison AJ, Trus BL, Cheng N, Steven AC, Watson MS (2005). A novel class of herpesvirus with bivalve hosts. J Gen Virol.

[11] Renault T (2024). Diseases of Bivalves. Historical and Current Perspectives.

[12] Abbadi M, Zamperin G, Gastaldelli M, Pascoli F, Rosani U (2018). Identification of a newly described OsHV-1 µvar from the North Adriatic Sea (Italy). J Gen Virol.

[13] Arzul I, Nicolas J-L, Davison AJ, Renault T (2001). French scallops: a new host for ostreid herpesvirus-1. Virology.

[14] Bai C-M, Morga B, Rosani U, Shi J, Li C (2019). Long-range PCR and high-throughput sequencing of Ostreid herpesvirus 1 indicate high genetic diversity and complex evolution process. Virology.

[15] Burioli EAV, Prearo M, Houssin M (2017). Complete genome sequence of Ostreid herpesvirus type 1 µVar isolated during mortality events in the Pacific oyster Crassostrea gigas in France and Ireland. Virology.

[16] Morga B, Jacquot M, Pelletier C, Chevignon G, Dégremont L (2021). Genomic diversity of the ostreid herpesvirus type 1 across time and location and among host species. Front Microbiol.

[17] Pelletier C, Chevignon G, Faury N, Arzul I, Garcia C (2025). Phylogenomic evidence for host specialization and genetic divergence in OsHV-1 infecting *Magallana gigas* and Ostrea edulis. Infect Genet Evol.

[18] Segarra A, Pépin JF, Arzul I, Morga B, Faury N (2010). Detection and description of a particular Ostreid herpesvirus 1 genotype associated with massive mortality outbreaks of Pacific oysters, *Crassostrea gigas*, in France in 2008. Virus Res.

[19] Xia J, Bai C, Wang C, Song X, Huang J (2015). Complete genome sequence of Ostreid herpesvirus-1 associated with mortalities of Scapharca broughtonii broodstocks. Virol J.

[20] Dégremont L (2013). Size and genotype affect resistance to mortality caused by OsHV-1 in *Crassostrea gigas*. Aquaculture.

[21] Petton B, Pernet F, Robert R, Boudry P (2013). Temperature influence on pathogen transmission and subsequent mortalities in juvenile Pacific oysters *Crassostrea gigas*. Aquacult Environ Interact.

[22] Renault T, Cochennec N, Rose-marie L, Bruno C (1994). Herpes-like virus infecting Japanese oyster (*Crassostrea gigas*) spat. Bull Eur Assoc Fish Pathol.

[23] Segarra A, Mauduit F, Faury N, Trancart S, Dégremont L (2014). Dual transcriptomics of virus-host interactions: comparing two Pacific oyster families presenting contrasted susceptibility to ostreid herpesvirus 1. BMC Genom.

[24] Degremont L, Benabdelmouna A (2014). Mortality associated with OsHV-1 in spat *Crassostrea gigas*: role of wild-caught spat in the horizontal transmission of the disease. Aquacult Int.

[25] Renault T, Tchaleu G, Faury N, Moreau P, Segarra A (2014). Genotyping of a microsatellite locus to differentiate clinical Ostreid herpesvirus 1 specimens. Vet Res.

[26] Divilov K, Wang X, Swisher AE, Yeoman PC, Rintoul M (2024). Ostreid herpesvirus 1 latent infection and reactivation in adult Pacific oysters, *Crassostrea gigas*. Virus Res.

[27] Evans O, Hick P, Whittington RJ (2017). Detection of Ostreid herpesvirus-1 microvariants in healthy Crassostrea gigas following disease events and their possible role as reservoirs of infection. J Invertebr Pathol.

[28] Schikorski D, Faury N, Pepin JF, Saulnier D, Tourbiez D (2011). Experimental ostreid herpesvirus 1 infection of the Pacific oyster *Crassostrea gigas*: kinetics of virus DNA detection by q-PCR in seawater and in oyster samples. Virus Res.

[29] Segarra A, Baillon L, Faury N, Tourbiez D, Renault T (2016). Detection and distribution of ostreid herpesvirus 1 in experimentally infected Pacific oyster spat. J Invertebrate Pathol.

[30] Lipart C, Renault T (2002). Herpes-like virus detection in infected *Crassostrea gigas* spat using DIG-labelled probes. J Virol Methods.

[31] Martenot C, Segarra A, Baillon L, Faury N, Houssin M (2016). In situ localization and tissue distribution of ostreid herpesvirus 1 proteins in infected Pacific oyster, *Crassostrea gigas*. J Invertebr Pathol.

[32] de Lorgeril J, Lucasson A, Petton B, Toulza E, Montagnani C (2018). Immune-suppression by OsHV-1 viral infection causes fatal bacteraemia in Pacific oysters. Nat Commun.

[33] Jouaux A, Lafont M, Blin J-L, Houssin M, Mathieu M (2013). Physiological change under OsHV-1 contamination in Pacific oyster *Crassostrea gigas* through massive mortality events on fields. BMC Genom.

[34] Morga B, Faury N, Guesdon S, Chollet B, Renault T (2017). Haemocytes from *Crassostrea gigas* and OsHV-1: a promising in vitro system to study host/virus interactions. J Invertebr Pathol.

[35] Renault T, Faury N, Barbosa-Solomieu V, Moreau K (2011). Suppression substractive hybridisation (SSH) and real time PCR reveal differential gene expression in the Pacific cupped oyster, *Crassostrea gigas*, challenged with Ostreid herpesvirus 1. Develop Comparat Immunol.

[36] Segarra A, Faury N, Pépin J-F, Renault T (2014). Transcriptomic study of 39 ostreid herpesvirus 1 genes during an experimental infection. J Invertebr Pathol.

[37] Green TJ, Raftos D, Speck P, Montagnani C (2015). Antiviral immunity in marine molluscs. J Gen Virol.

[38] Rosani U, Varotto L, Domeneghetti S, Arcangeli G, Pallavicini A (2015). Dual analysis of host and pathogen transcriptomes in ostreid herpesvirus 1-positive *Crassostrea gigas*. Environ Microbiol.

[39] Wang L, Song X, Song L (2018). The oyster immunity. Develop Comparat Immunol.

[40] Dégremont L (2011). Evidence of herpesvirus (OsHV-1) resistance in juvenile *Crassostrea gigas* selected for high resistance to the summer mortality phenomenon. Aquaculture.

[41] Dégremont L, Nourry M, Maurouard E (2015). Mass selection for survival and resistance to OsHV-1 infection in *Crassostrea gigas* spat in field conditions: response to selection after four generations. Aquaculture.

[42] Martenot C, Gervais O, Chollet B, Houssin M, Renault T (2017). Haemocytes collected from experimentally infected Pacific oysters, *Crassostrea gigas*: detection of ostreid herpesvirus 1 DNA, RNA, and proteins in relation with inhibition of apoptosis. PLOS ONE.

[43] Pepin JF, Riou A, Renault T (2008). Rapid and sensitive detection of ostreid herpesvirus 1 in oyster samples by real-time PCR. J Virol Methods.

[44] Saulnier D, De Decker S, Haffner P (2009). Real-time PCR assay for rapid detection and quantification of Vibrio aestuarianus in oyster and seawater: a useful tool for epidemiologic studies. J Microbiol Methods.

[45] Schikorski D, Renault T, Saulnier D, Faury N, Moreau P (2011). Experimental infection of Pacific oyster *Crassostrea gigas* spat by ostreid herpesvirus 1: demonstration of oyster spat susceptibility. Vet Res.

[46] Webb SC, Fidler A, Renault T (2007). Primers for PCR-based detection of ostreid herpes virus-1 (OsHV-1): application in a survey of New Zealand molluscs. Aquaculture.

[47] Rho M, Tang H, Ye Y (2010). FragGeneScan: predicting genes in short and error-prone reads. Nucleic Acids Res.

[48] Zhang K-Y, Gao Y-Z, Du M-Z, Liu S, Dong C (2019). Vgas: a viral genome annotation system. Front Microbiol.

[49] Jones P, Binns D, Chang H-Y, Fraser M, Li W (2014). InterProScan 5: genome-scale protein function classification. Bioinformatics.

[50] Cantalapiedra CP, Hernández-Plaza A, Letunic I, Bork P, Huerta-Cepas J (2021). eggNOG-mapper v2: functional annotation, orthology assignments, and domain prediction at the metagenomic scale. Mol Biol Evol.

[51] Song L, Florea L (2015). Rcorrector: efficient and accurate error correction for Illumina RNA-seq reads. Gigascience.

[52] Dobin A, Davis CA, Schlesinger F, Drenkow J, Zaleski C (2013). STAR: ultrafast universal RNA-seq aligner. Bioinformatics.

[53] Anders S, Pyl PT, Huber W (2015). HTSeq--a python framework to work with high-throughput sequencing data. Bioinformatics.

[54] Love MI, Huber W, Anders S (2014). Moderated estimation of fold change and dispersion for RNA-seq data with DESeq2. Genome Biol.

[55] Danecek P, Bonfield JK, Liddle J, Marshall J, Ohan V (2021). Twelve years of SAMtools and BCFtools. Gigascience.

[56] Quinlan AR, Hall IM (2010). BEDTools: a flexible suite of utilities for comparing genomic features. Bioinformatics.

[57] Wilcoxon F (1945). Individual comparisons by ranking methods on JSTOR. Biometrics Bulletin.

[58] Benjamini Y, Hochberg Y (1995). Controlling the false discovery rate: a practical and powerful approach to multiple testing. J R Stat Soc Series B Stat Methodol.

[59] Langfelder P, Horvath S (2008). WGCNA: an R package for weighted correlation network analysis. BMC Bioinformatics.

[60] Altschul SF (2014). Encyclopedia of Life Sciences.

[61] Bateman A, Martin MJ, Orchard S, Magrane M, Agivetova R (2021). UniProt: the universal protein knowledgebase in 2021. Nucleic Acids Res.

[62] Wright RM, Aglyamova GV, Meyer E, Matz MV (2015). Gene expression associated with white syndromes in a reef building coral, *Acropora hyacinthus*. BMC Genomics.

[63] Supek F, Bošnjak M, Škunca N, Šmuc T (2011). REVIGO summarizes and visualizes long lists of gene ontology terms. PLOS ONE.

[64] Honess RW, Roizman B (1974). Regulation of herpesvirus macromolecular synthesis. I. Cascade regulation of the synthesis of three groups of viral proteins. J Virol.

[65] Griffin DE, Knipe D, Howley P, Lamb R, Martin M (2001). Fields Virology.

[66] van Beurden SJ, Peeters BPH, Rottier PJM, Davison AJ, Engelsma MY (2013). Genome-wide gene expression analysis of anguillid herpesvirus 1. BMC Genomics.

[67] Cherrington JM, Khoury EL, Mocarski ES (1991). Human cytomegalovirus ie2 negatively regulates alpha gene expression via a short target sequence near the transcription start site. J Virol.

[68] Harkness JM, Kader M, DeLuca NA (2014). Transcription of the herpes simplex virus 1 genome during productive and quiescent infection of neuronal and nonneuronal cells. J Virol.

[69] Rozman B, Nachshon A, Levi Samia R, Lavi M, Schwartz M (2022). Temporal dynamics of HCMV gene expression in lytic and latent infections. Cell Rep.

[70] Tombácz D, Tóth JS, Petrovszki P, Boldogkoi Z (2009). Whole-genome analysis of pseudorabies virus gene expression by real-time quantitative RT-PCR assay. BMC Genomics.

[71] Bachère E, Chagot D, Grizel H (1988). Separation of crassostrea gigas. Develop Comparat Immunol.

[72] de la Ballina NR, Maresca F, Cao A, Villalba A (2022). Bivalve haemocyte subpopulations: a review. Front Immunol.

[73] Meng J, Zhang G, Wang W-X (2022). Functional heterogeneity of immune defenses in molluscan oysters *Crassostrea hongkongensis* revealed by high-throughput single-cell transcriptome. Fish Shellfish Immunol.

[74] Wang W, Li M, Wang L, Chen H, Liu Z (2017). The granulocytes are the main immunocompetent hemocytes in Crassostrea gigas. Develop Comparat Immunol.

[75] Alharshawi K, Cox B, Ariza ME (2022). Examination of control asymptomatic cohorts reveals heightened anti-EBV and HHV-6 A/B dUTPase antibodies in the aging populations. J Med Virol.

[76] Leopardi R, Roizman B (1996). The herpes simplex virus major regulatory protein ICP4 blocks apoptosis induced by the virus or by hyperthermia. Proc Natl Acad Sci USA.

[77] Zarrouk K, Piret J, Boivin G (2017). Herpesvirus DNA polymerases: structures, functions and inhibitors. Virus Res.

[78] Crump C (2018). Virus assembly and egress of HSV. Adv Exp Med Biol.

[79] Leprêtre M, Faury N, Segarra A, Claverol S, Degremont L (2020). Comparative proteomics of ostreid herpesvirus 1 and Pacific oyster interactions with two families exhibiting contrasted susceptibility to viral infection. Front Immunol.

[80] Rosani U, Bortoletto E, Zhang X, Huang B-W, Xin L-S (2024). Long-read transcriptomics of Ostreid herpesvirus 1 uncovers a conserved expression strategy for the capsid maturation module and pinpoints a mechanism for evasion of the ADAR-based antiviral defence. Virus Evol.

[81] Volkening JD, Spatz SJ, Ponnuraj N, Akbar H, Arrington JV (2023). Viral proteogenomic and expression profiling during productive replication of a skin-tropic herpesvirus in the natural host. PLOS Pathog.

[82] Khorkova O, Hsiao J, Wahlestedt C (2015). Basic biology and therapeutic implications of lncRNA. Adv Drug Deliv Rev.

[83] Zhang X, Cui Y, Ding X, Liu S, Han B (2021). Analysis of mRNA‑lncRNA and mRNA‑lncRNA-pathway co‑expression networks based on WGCNA in developing pediatric sepsis. Bioengineered.

[84] Carter KL, Ward PL, Roizman B (1996). Characterization of the products of the U(L)43 gene of herpes simplex virus 1: potential implications for regulation of gene expression by antisense transcription. J Virol.

[85] Jacquemont B, Roizman B (1975). RNA synthesis in cells infected with herpes simplex virus. X. Properties of viral symmetric transcripts and of double-stranded RNA prepared from them. J Virol.

[86] Kozak M, Roizman B (1975). RNA synthesis in cells infected with herpes simplex virus. IX. Evidence for accumulation of abundant symmetric transcripts in nuclei. J Virol.

[87] Zhang G, Raghavan B, Kotur M, Cheatham J, Sedmak D (2007). Antisense transcription in the human cytomegalovirus transcriptome. J Virol.

[88] Bai H, Jiang L, Wang X, Gao X, Bing J (2019). Transcriptomic analysis of mouse cochleae suffering from gentamicin damage reveals the signalling pathways involved in hair cell regeneration. Sci Rep.

[89] Ryu AH, Eckalbar WL, Kreimer A, Yosef N, Ahituv N (2017). Use antibiotics in cell culture with caution: genome-wide identification of antibiotic-induced changes in gene expression and regulation. Sci Rep.

[90] Mello DF, Trevisan R, Even Y, Foulon V, Lambert C (2025). In vitro dynamics of oyster hemocytes in plasma-based primary cultures. Exp Cell Res.

[91] Mello DF, Trevisan R, Even Y, Foulon V, Lambert C Establishment of a long-term primary culture of oyster hemocytes and novel insights about their function, metabolism, and behavior. SSRN.

